# Theoretical framework for engineering Boltzmann luminescent nanothermometry

**DOI:** 10.1038/s41377-026-02333-2

**Published:** 2026-05-21

**Authors:** Mingzhu Yang, Hongxin Zhang, Fan Zhang

**Affiliations:** Laboratory of Advanced Materials, College of Smart Materials and Future Energy, Department of Chemistry, New Cornerstone Science Laboratory, State Key Laboratory of Molecular Engineering of Polymers, Shanghai Key Laboratory of Molecular Catalysis and Innovative Materials, Fudan University, Shanghai, China

**Keywords:** Nanoparticles, Biomaterials

## Abstract

Luminescent nanothermometry based on thermally coupled levels (TCLs) has emerged as a powerful tool for non-invasive temperature sensing, but it still lacks sufficient theoretical guidelines. To address this issue, a theoretical framework for Boltzmann luminescent nanothermometry has been established, which quantitatively defines the temperature window for establishing thermal equilibrium in TCLs, establishes a practical criterion for stable thermal coupling of TCLs, and enables predictive material design of temperature sensitivity. Based on this framework, a high sensitivity of 6.17% K^-1^ is achieved, providing a theoretical basis for the rational design of high-precision nanothermometers.

**Figure:**
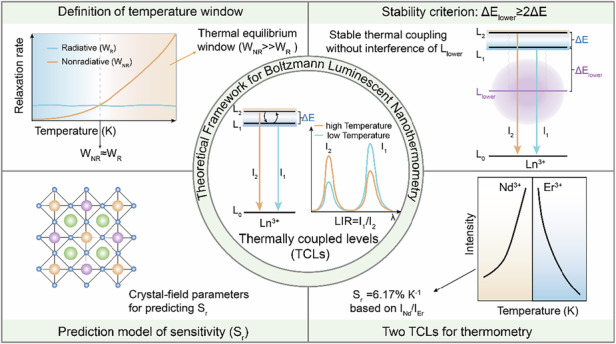
A theoretical framework for TCLs-based luminescent nanothermometry defines the thermal equilibrium window, establishes a stability criterion and guides rational predictive design

A theoretical framework for TCLs-based luminescent nanothermometry defines the thermal equilibrium window, establishes a stability criterion and guides rational predictive design

Luminescent nanothermometry based on temperature-sensitive optical materials has emerged as a powerful tool for non-invasive, fast-response and high-resolution temperature sensing, showing great potential in different fields, including nanofluidics, microelectronics and biomedicine^1,2^. Among various techniques, ratiometric thermometry based on thermally coupled levels (TCLs) of lanthanide ions is particularly attractive. Their relative population of two closely spaced excited states follows the Boltzmann distribution, making their luminescence intensity ratio (LIR) a self-referencing and environmentally robust temperature indicator (Fig. 1, central panel)^3^. Boltzmann luminescent nanothermometry based on this principle has enabled a wide array of applications, from mapping temperature gradients at the sub-cellular level to providing real-time thermal feedback during in vivo photothermal therapy^4,5^. However, deviations between the experimental observations and the ideal Boltzmann behavior of TCLs are frequently reported^6^. Moreover, key operational parameters such as the temperature window for thermal coupling are often determined empirically, lacking a unified quantitative definition^7^. This critical gap severely restricts the practical application and standardization of TCLs-based luminescent nanothermometry.

**Fig. 1:**
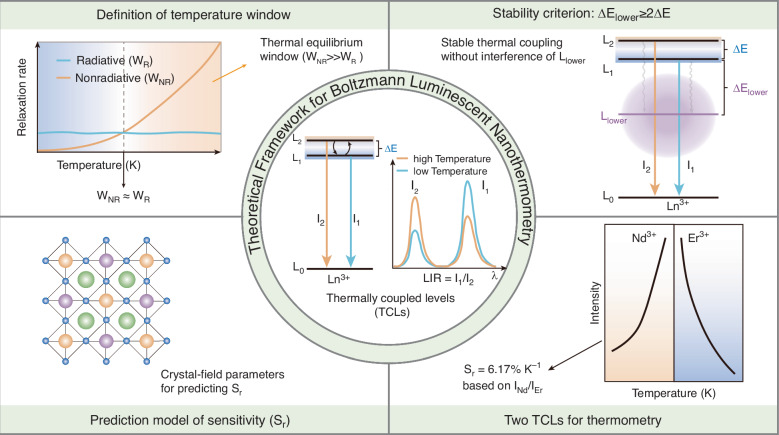
Schematic illustration of the theoretical framework for Boltzmann luminescent nanothermometry. Central panel: Fundamental principle of thermally coupled levels (TCLs) in lanthanide ions (Ln^3+^). Top left: Definition of the temperature window. The competition between the radiative relaxation rate (W_R_) and nonradiative relaxation rate (W_NR_) defines a temperature-critical region where thermal equilibrium is established. Top right: The stability criterion for Boltzmann coupling. To ensure stable thermal coupling without interference of the nearest lower level (L_lower_), the energy gap to the L_lower_ should satisfy ΔE_lower_ ≥ 2ΔE. Bottom left: Prediction model for relative sensitivity (S_r_) through crystal-field parameters. Bottom right: High-sensitivity thermometry enabled by combining two TCLs from Nd^3+^ and Er^3+^, respectively

Recent years have witnessed deepening insights into the origins of these discrepancies. On one hand, factors such as the thermal distribution of Stark sublevels, interference from parasitic nonradiative relaxation channels, and non-thermal contributions to the upper-level population have been successively revealed, providing important foundations for understanding theory-experiment mismatches^8,9^. On the other hand, researchers have begun to explore the external conditions required for TCLs to function effectively, establishing empirical temperature windows for different thermalization energy gap (ΔE) of TCLs and investigating how factors such as host phonon energy, lanthanide-ligand distance, and transition type, influence the onset temperature of thermal equilibrium^10^. These works have explained why deviations occur from different perspectives and preliminarily explored how to optimize performance through material selection. However, the fundamental rules governing TCLs formation and the reliable prediction of relative sensitivity (S_r_) in specific hosts remain unclear.

In a recent study published in *Light: Science & Applications*, Fu et al. address these challenges by establishing a comprehensive theoretical framework and predictive design principles for TCLs-based nanothermometry (Fig. 1)^11^. This work makes three theoretical advances. Firstly, it clarifies the temperature window for effective thermal coupling. By analyzing the competition between nonradiative relaxation rates (W_NR_) and radiative rates (W_R_), they define the temperature-critical region, quantifying the previously vague concept of thermal equilibrium and explaining why conventional TCLs struggle at low temperatures (Fig. 1, top left). Secondly, this work reveals the interference effect of the nearest lower level (L_lower_) on the thermal equilibrium of TCLs and proposes a practical criterion. Through theoretical derivation and systematic simulations, they establish a quantitative criterion: the energy gap to the L_lower_ must satisfy ΔE_lower_ ≥ 2ΔE for this interference to be negligible (Fig. 1, top right). This criterion provides a practical rule for pre-screening potential TCLs, explaining why lanthanide ions possess numerous adjacent energy levels within the 200–2000 cm^−1^ energy gap range, but only a limited number of these pairs exhibit reliable Boltzmann behavior. Thirdly, it establishes a quantitative link between macroscopic sensitivity and microscopic material parameters. A splitting factor K_e_ to quantitatively correlate the thermal coupling gap ΔE with crystal-field related parameters is introduced, enabling them to derive a predictive formula for the relative sensitivity S_r_ (Fig. 1, bottom left). For given TCLs, the host-determined intrinsic ΔE provides a criterion for material selection, meaning that researchers can now predict temperature sensing performance before material synthesis, advancing TCLs thermometry from empirical exploration to rational design.

Building on these theoretical insights, a two TCLs combination strategy is proposed to achieve high sensitivity. By selecting the thermally enhanced TCLs of Nd^3+^ and the thermally quenched TCLs of Er^3+^, a high relative sensitivity of 6.17% K^-1^ at 313 K in the LiYF_4_:Yb,Nd,Er system is achieved (Fig. 1, bottom right). This value significantly exceeds that of conventional single-TCLs thermometry, which faces a theoretical limit on S_r_ (typically <3% K^-1^ at room temperature) due to the upper bound of ΔE ( ~ 2000 cm^−1^)^12^. It also exhibits superior performance compared to recently reported non-thermal coupling thermometers^13^. Furthermore, flexible ultrathin thermosensing patches are fabricated for non-contact in situ temperature monitoring, demonstrating the potential for real-time and high-precision thermal readout in complex environments.

In summary, Fu et al. establish clear mechanistic criteria and predictive design rules for Boltzmann luminescent nanothermometry, providing a complete framework for the rational design of high-precision luminescent thermometers^11^. Looking forward, based on the rational design framework established by Fu et al., several promising directions can be further explored to promote the application of luminescent nanothermometry. Firstly, extending the design strategy to other luminescent ion systems beyond Ln^3+^. Although the current work focuses on lanthanide TCLs, transition metal ions (e.g., Cr^3+^) also offer promising opportunities for Boltzmann thermometry. Transition metal ions possess distinct electron-phonon coupling characteristics and benefit from higher brightness due to stronger absorption cross-sections of d-d transitions^14^. Their thermal coupling behavior suggests that systematic exploration of transition metal ions with suitable energy level structures and strong electron-phonon coupling could yield new thermometers with unprecedented dynamic ranges and brightness. Secondly, developing thermometers emitting in the second near-infrared window (NIR-II, 1000–2000 nm)^15,16^. Combining the design principles with NIR-II emissions of Yb^3+^, Er^3+^, Tm^3+^, etc., to develop luminescent nanothermometers with both high sensitivity and deep tissue penetration will substantially advance the practical application of luminescence thermometry in biomedicine.
